# Supplementary material for: DMRforPairs: identifying Differentially Methylated Regions between unique samples using array based methylation profiles
Source: BMC Bioinformatics. 2014 May 15;15:141. doi: 10.1186/1471-2105-15-141 (PMC4046028; doi:10.1186/1471-2105-15-141)
Supplement: Additional file 2 — DMRforPairs output for the comparison of A431-MCF7 and NA17018-NA17105. Please start from the HTML files in each folder. Available via the BMC Bioinformatics website. [file 1471-2105-15-141-S2.zip › 1394847754114233_MOESM2_ESM/A431_MCF7/figures/24189.pdf]

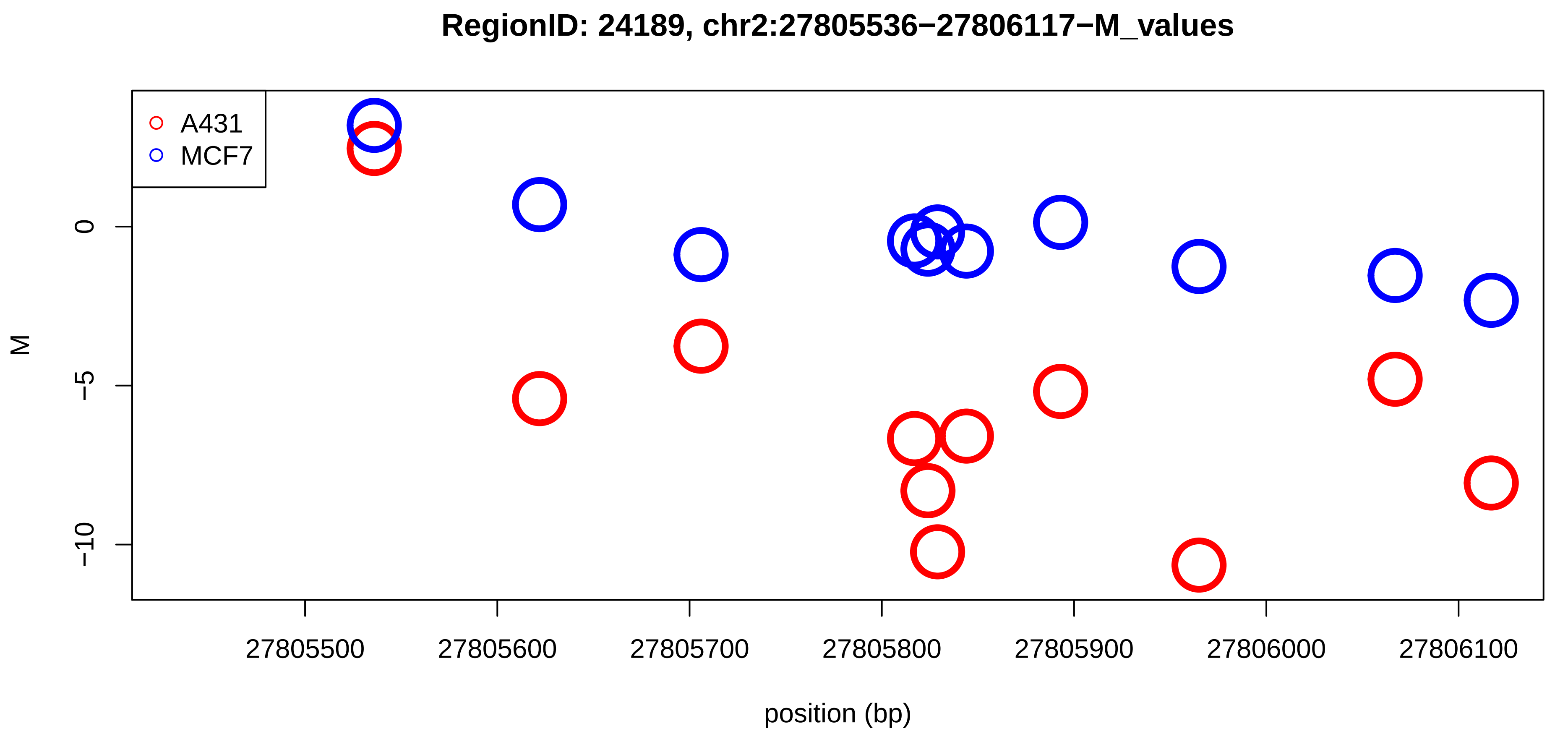
RegionID: 24189, chr2:27805536−27806117−M_values
A431
MCF7
M
0
−5
−10
27805500
27805600
27805700
27805800
27805900
27806000
27806100
position (bp)

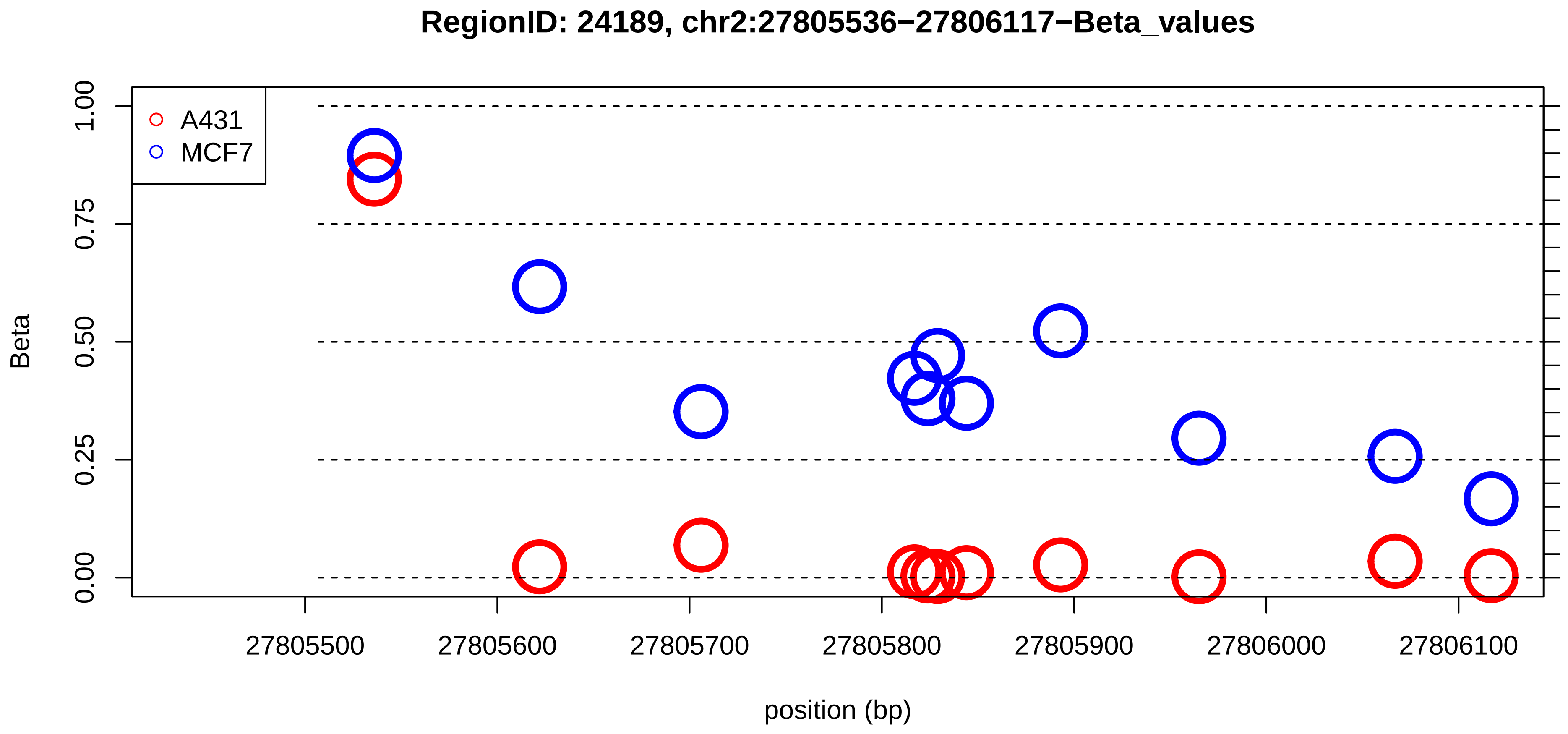
RegionID: 24189, chr2:27805536−27806117−Beta_values
A431
MCF7
Beta
0.00
0.25
0.50
0.75
1.00
27805500
27805600
27805700
27805800
27805900
27806000
27806100
position (bp)

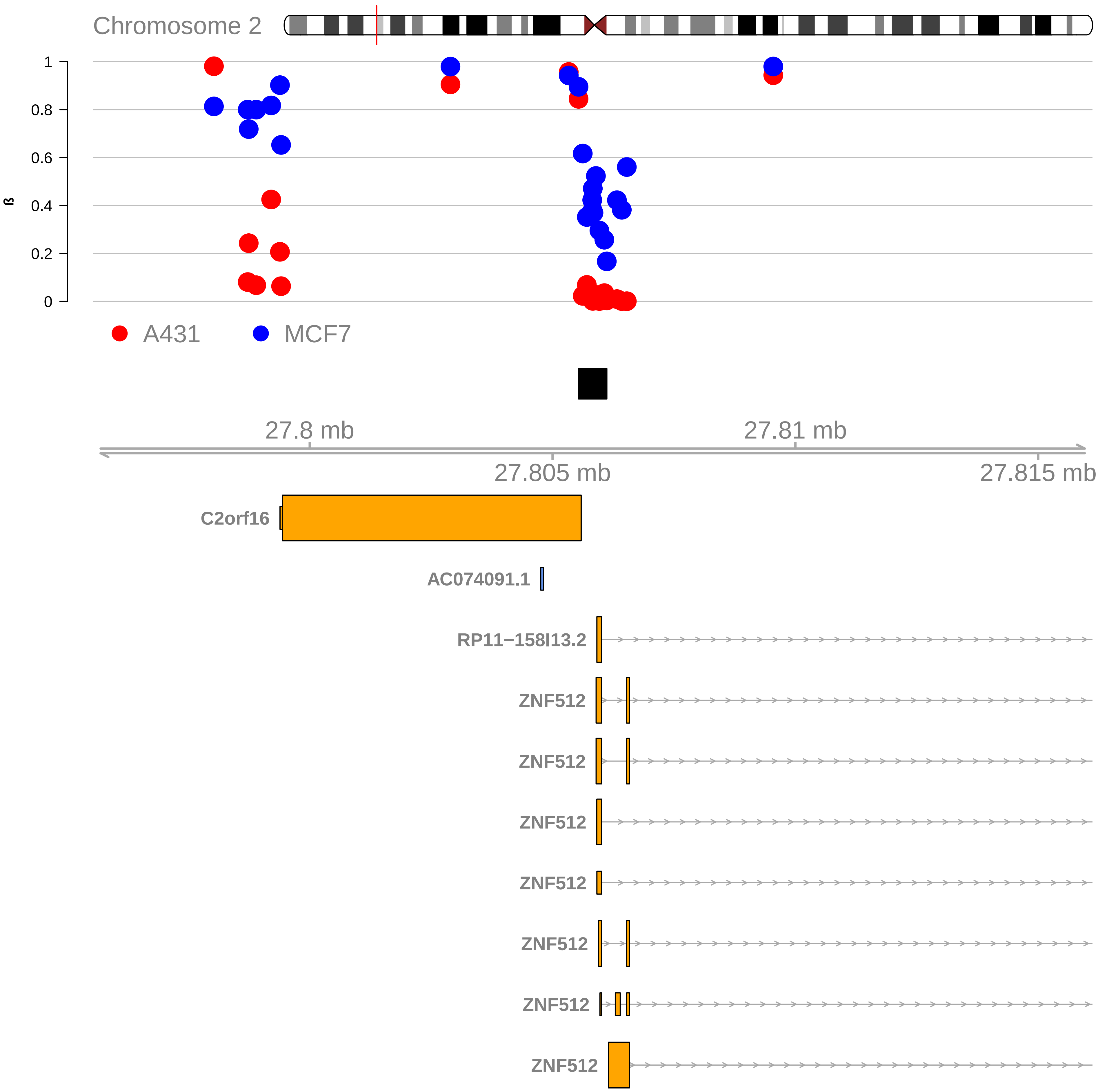

Chromosome 2
1
0.8
0.6
0.4
0.2
0
ß
A431
MCF7
27.8 mb
27.81 mb
27.805 mb
27.815 mb
C2orf16
AC074091.1
RP11−158I13.2
ZNF512
ZNF512
ZNF512
ZNF512
ZNF512
ZNF512
ZNF512
